# Metabolomic analysis of the effects of a mixed culture of *Saccharomyces cerevisiae* and *Lactiplantibacillus plantarum* on the physicochemical and quality characteristics of apple cider vinegar

**DOI:** 10.3389/fnut.2023.1142517

**Published:** 2023-03-14

**Authors:** Ya-Nan Li, Yue Luo, Zhen-Ming Lu, Yan-Lin Dong, Li-Juan Chai, Jin-Song Shi, Xiao-Juan Zhang, Zheng-Hong Xu

**Affiliations:** ^1^Key Laboratory of Industrial Biotechnology of Ministry of Education, School of Biotechnology, Jiangnan University, Wuxi, China; ^2^National Engineering Research Center of Cereal Fermentation and Food Biomanufacturing, Jiangnan University, Wuxi, China; ^3^Apple Cider Vinegar Engineering and Technology Research Center of Yantai, Lvjie Co., Ltd., Yantai, China; ^4^Jiangsu Engineering Research Center for Bioactive Products Processing Technology, Jiangnan University, Wuxi, China; ^5^School of Life Sciences and Health Engineering, Jiangnan University, Wuxi, China

**Keywords:** mixed-culture fermentation, metabolomics, apple cider vinegar, physicochemical characteristics, quality traits

## Abstract

**Introduction:**

This study compared differences in physicochemical characteristics of the vinegar made by a mixed culture (MC) of *Saccharomyces cerevisiae* and *Lactiplantibacillus plantarum* and a pure culture (PC) of *Saccharomyces cerevisiae*.

**Methods:**

The fermentation process was monitored, and metabolomics analysis by Liquid Chromagraphy-Mass Spectrometry (LC-MS) was applied to the compositional differences between PC and MC vinegars, combined with quantification of organic acids, amino acids and B vitamins.

**Results:**

A total of 71 differential metabolites including amino acids, organic acids and carbohydrates, and six possible key metabolic pathways were identified. MC enhanced the malic acid utilization and pyruvate acid metabolism during fermentation, increasing substrate-level phosphorylation, and supplying more energy for cellular metabolism. Higher acidity at the beginning of acetic acid fermentation, resulting from lactic acid production by *Lactiplantibacillus plantarum* in MC, suppressed the cellular metabolism and growth of *Acetobacter pasteurianus*, but enhanced its alcohol metabolism and acetic acid production in MC. MC vinegar contained more vitamin B, total flavonoids, total organic acids, amino acids and had a higher antioxidant capacity. MC enhanced the volatile substances, particularly ethyl lactate, ethyl caprate and ethyl caproate, which contributed to a stronger fruity aroma.

**Discussion:**

These results indicated the mixed culture in alcoholic fermentation can effectively enhance the flavor and quality of apple cider vinegar.

## 1. Introduction

Apple cider vinegar is a popular consumer product with a long history of use, and is consumed for multiple purposes, including as a condiment, soft drink and as a natural remedy/health product. Apple cider vinegar is produced by two-stage submerged fermentation of apple juice (alcoholic fermentation by *Saccharomyces cerevisiae*, followed by acetic acid fermentation by *Acetobacter pasteurianus*).

Apple cider vinegar is rich in amino acids, organic acids, phenolic acids, and flavonoids (1–5). Some of these compounds are associated with health benefits and contribute to antioxidant capacity, antimicrobial, and immunostimulatory activity (4–7). The potential health benefits of apple cider vinegar have attracted increasing research interest. Apple cider vinegar reduces blood triglyceride levels in mice (8) and has beneficial effects on blood sugar, insulin resistance and oxidative stress in patients with diabetes and dyslipidemia (9). The organoleptic qualities of vinegar are determined by both its volatile and non-volatile components. The dominant flavor component of apple cider vinegar is acetic acid, a volatile and pungent organic acid, resulting in high astringency and sourness; these sensory properties limit consumer acceptance for natural health purposes. Minor taste and flavor components also contribute strongly to the sensory attributes of vinegar. Non-volatile organic acids such as malic, citric, succinic and lactic acids may reduce astringency, but increase sourness. Amino acids contribute sweetness, bitterness and umami to the taste. and the bioconversion of these amino acids during vinegar fermentation affects the overall taste. The volatile compound profile of vinegar is a key determinant of its organoleptic properties. Phenylethanol, octanoic acid, ethyl acetate, butyl acetate, and isoamyl alcohol are key compounds contributing to the floral aroma of apple cider vinegar (2, 10). The final quality and sensory properties of apple cider vinegar are determined by the chemical complexity generated by the fermentation process used. Most industrial submerged vinegar fermentation is produced by a two-stage culture liquid fermentation, which involves alcoholic fermentation, the anaerobic conversion of fermentable sugars to ethanol by yeast (usually *S. cerevisiae*), followed by acetic acid fermentation, the aerobic oxidation of ethanol to acetic acid (usually by *A. pasteurianus* or undefined starter cultures of acetic acid bacteria) (11). Solid-state fermentation of grains, or natural liquor-state fermentation (surface fermentation) with multiple microbial strains produces greater diversity of metabolites and more varied sensory properties. For example, although the processes and raw materials of Chinese cereal vinegar and sherry vinegar are markedly different, they are both produced by multi-strain, collaborative fermentation, and lactic acid bacteria are important fermentation strains, with high abundance and activity (12–14). The relative abundance of *L. plantarum* was reported as 33.9 and 88.6% in sherry vinegar and Zhenjiang aromatic vinegar fermentation, respectively (12, 13). *Lactiplantibacillus plantarum* and *S. cerevisiae* are found in many natural fermentation systems, such as wine and kefir. The [GAR + ] Prion Induction ability of lactic acid bacteria can establish a mutually beneficial relationship with *S. cerevisiae* during wine fermentation. During kefir fermentation, *Lactobacilli* and *yeasts* can promote each other’s viability through nutrient and metabolite exchange (15–19). Therefore, considering the frequently observed positive interactions between these two species, incorporation of *Lactobacillus* in the alcoholic fermentation stage of vinegar production has potential to enrich the flavor compound composition and organoleptic properties of vinegar.

Fermentation of Mahali cherries by yeast and *Lactobacilli* reduced the sourness of the fruit (20). Addition of *Lactobacilli* to the alcoholic fermentation stage of citrus vinegar improved its antioxidant properties, organic acid, flavor ester and alcohol content, umami taste and sweet amino acid content, thereby improving its flavor and quality (6). Multi-strain vinegar fermentation has clear potential to improve cider vinegar quality, but there is little information on differences from single-strain fermentation at the metabolomic level, or the associated bioconversion pathways responsible for the differences.

Previously, we comprehensively investigated the microbiome of Chinese solid-state fermented cereal vinegar, and isolated *L. plantarum* F from the alcoholic fermentation stage of Chinese traditional cereal vinegar, which has good fermentation capacity and alcohol tolerance in both liquid and solid-state vinegar fermentation. In this study, apple cider vinegar co-fermented by *L. plantarum* F and *S. cerevisiae* in alcoholic fermentation stage was compared with that fermented by single-strain *S. cerevisiae*. Dynamic fermentation parameters were collected, combined with liquid chromatography-tandem mass spectrometry (LC-MS) based metabolomics analysis and quantification of amino acids, vitamins and other physicochemical characteristics. The effects of mixed culture (MC) and pure culture (PC) on cell growth, and quality traits of apple cider vinegar were determined, to elucidate the important metabolic pathways in the two fermentation systems. The volatile compound profiles were also compared to identify differential volatiles between the two fermentation systems. This study will deepen understanding of the metabolic mechanisms of multi-strain collaborative vinegar fermentation.

## 2. Materials and methods

### 2.1. Chemicals and materials

Apple juice was from Shandong Lvjie (China). *L. plantarum* F, *S. cerevisiae* R and *A. pasteurianus* X were from our lab culture collection. Standards of amino acids, organic acids, and n-pentane (C_5_H_12_) were from Sigma-Aldrich (St. Louis, MO, USA). All other chemicals and solvents were from Sinopharm Chemical Reagents (Shanghai, China). The ATP assay kit was from Jiancheng (Nanjing, China).

### 2.2. Screening strain

All strains were isolates obtained from vinegar pei during the fermentation process of traditional cereal vinegar. The detailed isolation procedure were described in Supplementary Information (Note 1).

### 2.3. Culture medium

YPD medium (yeast extract 10 g/L, glucose 20 g/L, peptone 20 g/L) was used for fermentation of *S. cerevisiae* R. MRS medium (peptone 10 g/L, yeast extract 5 g/L, ammonium citrate 2 g/L, glucose 20 g/L, dipotassium phosphate 2 g/L, magnesium sulfate 0.58 g/L, manganese sulfate 0.28 g/L, beef extract 10 g/L, tween-80 1 mL) was used for fermentation of *L. plantarum* F. LB medium (yeast extract 10 g/L, glucose 20 g/L, ethanol 2%) was used for fermentation of *A. pasteurianus* X.

### 2.4. Fermentation of cider and vinegar

Submerged fermentation was conducted by shake-flasks in this study. Apple juice was adjusted to 12°Brix. For the alcoholic fermentation stage, the apple juice was incubated for 5 days at 30°C with *S. cerevisiae* R (10^6^ CFU/mL, 2% v/v) and *L. plantarum* F (10^6^ CFU/mL, 2% v/v) for MC. For PC, the apple juice was incubated for 5 days at 30°C with *S. cerevisiae* R alone (10^6^ CFU/mL, 2% v/v). When an alcoholic fermentation was complete, the cells were removed by centrifugation at 3,214 *g* and the supernatant was inoculated with 2–4% *A. pasteurianus* X seed culture (OD_600_ 0.8), then incubated on a rotary shaking incubator (180 rpm) for 5 days at 30°C. When an acetic acid fermentation was complete, the cells were removed by centrifugation at 3,214 *g* and the supernatant was retained. All the fermentations were carried out independently in triplicate.

### 2.5. Analysis of the pH, soluble solids, reducing sugar, ethanol, acetic acid, ATP, ADH, ALDH, and cell growth

The pH was measured by FE28-Standard pH meter (Mettler-Toledo, Greifensee, Switzerland). The soluble solids content was determined at 20°C using RA-250WE refractometer (KEM, Kyoto, Japan) and reported as Brix. Ethanol was determined by gas chromatography (GC). The reducing sugar content was measured by the 3,5-dinitrosalicylic acid (DNS) method The number of colony forming units (CFU) per mL of *L. plantarum* F and *S. cerevisiae* R was counted according to the dilution factor and the number of colonies on plates with 30–300 colonies after incubation at 37 and 30°C, respectively. Biomass of *A. pasteurianus* X was quantified by OD_600_. The enzyme activities of alcohol dehydrogenase (ADH) and aldehyde dehydrogenase (ALDH) in cells and fermentation medium were determined by a colorimetric method, as described previously (2). ATP content was determined by an ATP assay kit (colorimetric method). Results for metabolite production are expressed as grams per cell unit (g/U). One cell unit is defined as 10^9^ cells (*S. cerevisiae* R and *L. plantarum* F) or cell suspension with an optical density of 1 at 600 nm (*A. pasteurianus* X).

### 2.6. Antioxidant capacity

DPPH radical scavenging capacity was determined as described previously (21, 22). The ABTS radical scavenging capacity was determined as described previously (23, 24), with minor modifications. The ferric reducing antioxidant power (FRAP) was determined as described previously (25, 26), with minor modifications.

### 2.7. Total flavones, total phenolics, and water-soluble vitamin B

The total flavonoid content was determined as described previously (24, 27). The total phenolic content of the samples was determined using Folin-Ciocalteau reagent, with gallic acid as standard, as described previously (24, 28). Water-soluble vitamin B was determined as described previously (25). The conditions were as follows: column, Alltima C18 (250 mm × 4.6 mm, 5 μ^4^); mobile phase, 50 mmolL^–1^ ammonium dihydrogen phosphate solution (phosphoric acid adjusted to pH 3.0)-acetonitrile (95:5); flow rate, 0.5 mLmin^–1^; detection wavelength, 275 nm (detection of vitamin B1, vitamin B6, nicotinamide B3) and 210 nm (detection of pantothenic acid B5); column temperature, room temperature; injection volume, 20 μm.

### 2.8. Organic acid and free amino acid concentrations

Organic acids were determined by HPLC (Shimadzu, Tokyo, Japan) as described previously (29, 30). The organic acid was determined using 0.01 mol/L KH_2_PO_4_-H_3_PO_4_ (pH 2.7) and methanol as the mobile phase, and isocratic at a ratio of 97:3 mode for elution. The flow rate was 0.7 mL/min, and the UV–Vis spectrum detection was set to 210 nm. Standard solutions of different concentrations were prepared of oxalic acid, tartaric acid, malic acid, lactic acid, acetic acid, oxaloacetate, isocitrate, *cis*-aconitate, citric acid, and succinic acid and mixed for HPLC analysis. Each compound was quantified by comparing the peak area to the standard curve generated for that compound.

The free amino acid composition was determined with an Agilent 1,100 amino acid analyzer (Agilent Technologies, Santa Clara, CA, USA), as described previously (6, 31), with minor modifications. The free amino acid was determined using An Agilent Zorbax SB-C18 column (4.6 mm ID × 60 mm; Agilent Technologies, Palo Alto, CA, USA) was used, and the flow rate was 0.7 mL/min. The column temperature was 45°C; detector: Ex 340 nm, Em 450 nm; mobile phase A: 20 mmol sodium acetate solution; and the flow rate was 0.50 mL/min. Meanwhile, mobile phase B involved 20 mmol sodium acetate-methanol-acetonitrile (1:2:2, (v/v/v), and the flow rate was 0.4 mL/min). Each amino acid was quantified using the calibration curve of the corresponding authentic standard.

### 2.9. GC-MS analysis of volatile compounds by solid phase micro-extraction (SPME)

Volatile compounds were determined by GC-MS as described previously (6, 25) with minor modifications. NaCl (1 g) was added to a 15 mL glass vial, followed by the sample (6 mL) and 2-methyl-2-butanol (10 μL, 8 g/L, Sigma-Aldrich) as the internal standard, then the vial was pre-equilibrated for 15 min at 45°C. The SPME fiber was inserted into the head space and maintained for 40 min at 45°C. The analyses were conducted on a GC-MS system (Trace GC-1310-ISQ LT; Thermo Finnigan, Austin, TX, USA) equipped with the DB-WAX column (30 m × 0.25 mm, 0.25 μm; Agilent). The GC oven temperature was initially 40°C, increased to 230°C at 5°C/min and held constant for 8 min. Helium was used as the carrier gas at a flow rate of 1.2 mL/min. Quantitation of volatile compounds was based on the internal standard (2-Methyl-2-butanol). Mass spectra were generated in the electron impact (EI) mode at 70 eV. The spectrum was measured over the m/z range of 40–400, and the solvent elution delay time was 4 min.

### 2.10. Metabolomics analysis by LC-MS

All samples thawed at 4°C, and then 1 μL from each sample is transferred in a 2 mL centrifuge tube. Next, 400 μL methanol was added to each tube (−20°C) and swirled for 60 s. After centrifugation at 12,000 rpm for 10 min at 4°C, the supernatant was taken and transferred to a new 2 mL centrifuge tube for vacuum concentration and drying. The supernatant (samples to be tested) was obtained by resolution of 150 μL 2-chlorophenylalanine (4 ppm) 80% methanol solution, which was filtered by 0.22 μm membrane. Each sample was taken 20 μL and mixed into QC sample, and LC-MS was used for detection.

Six biological replicates were performed for each experiment. The ESI-MSn analyses were performed on a Thermo Q Exactive Plus mass spectrometer with spray voltages of 3.5 and-2.5 kV in the positive and negative modes, respectively. Sheath gas and auxiliary gas were set at 30 and 10 arbitrary units, respectively. The capillary temperature was 325°C. The analyzer scanned over a mass range of m/z 81–1,000 full scan at a mass resolution of 70,000. Data dependent acquisition (DDA) MS/MS experiments were performed with HCD scanning. The normalized collision energy was 30 eV. Dynamic exclusion was implemented to remove some unnecessary information in MS/MS spectra. More detail can be found in previous reports (32–34).

The obtained original data was converted into mzXML format (xcms input file format) by Proteowizard software (v3.0.8789). The XCMS package R (v3.3.2) was used to filtrate peaks identification, peaks filtration and peaks alignment. The main parameters are bw = 2, ppm = 15, peakwidth = c (5, 30), mzwid = 0.015, mzdiff = 0.01, method = centWave. The data matrix including mass to charge ratio (m/z), retention time (rt) and intensity was obtained. For positive ion mode, 10,049 precursor molecules have been acquired, while for negative ion mode, 8,908 precursor molecules have been acquired, and the data will be exported to excel for subsequent analysis. In order to enable comparison of data of different orders of magnitude, batch normalization of peak area was performed on the data.

### 2.11. Sensory evaluation

Sensory evaluation of apple cider vinegar was performed as described previously (6) with minor modifications. The sensory panel consisted of 15 trained individuals (aged 22–40) from the National Engineering Research Center of Cereal Fermentation and Food Biomanufacturing, Jiangnan University, China. The sensory descriptors agreed by the team members were acidic, ethanolic, flowery, fruity and burnt aromas, and sour, umami, sweet, salty, and bitter tastes. Fifteen panelists were selected and trained in the sensory room at room temperature (23 ± 2°C). The panelists scored each attribute on a linear scale from 0 (lowest intensity) to 9 (highest intensity). Water was provided to the panelists to cleanse the palate. The training and evaluation were organized according to the International Organization for Standardization (35) and conducted in a sensory laboratory that complies with the American Society for Testing and Materials criteria. The study was conducted according to the guidelines of the Declaration of Helsinki and approved by the Medical Ethics Committee of Jiangnan University.

### 2.12. Data analysis

The metabolites were identified using the HMDB^1^ and METLIN^2^ metabolomic data bases. Multivariate statistical analysis including PCA and (orthogonal) projection to PLS-DA were then performed using SIMCA-P + version 14.0 software (Umetrics, Umeå, Sweden) to distinguish the overall differences in metabolic profiles between groups. In the PLS-DA analysis, a variable importance in projection (VIP) score >1.0 was considered a differential variable, and in the *t*-tests, the variables with *p* < 0.05 were considered significant (36). The metabolites with VIP >1 and *p* < 0.05 were selected as differential metabolites.

All experiments were carried out in triplicate, and each sample was analyzed in triplicate. Analysis of Variance (*T* test) followed by using SPSS software version 25.0 (SPSS-IBM Chicago, IL, USA). Differences were considered statistically significant at *p* < 0.05.

## 3. Results and discussion

### 3.1. The effect of mixed culture on physicochemical properties of apple cider vinegar

**FIGURE 1:**
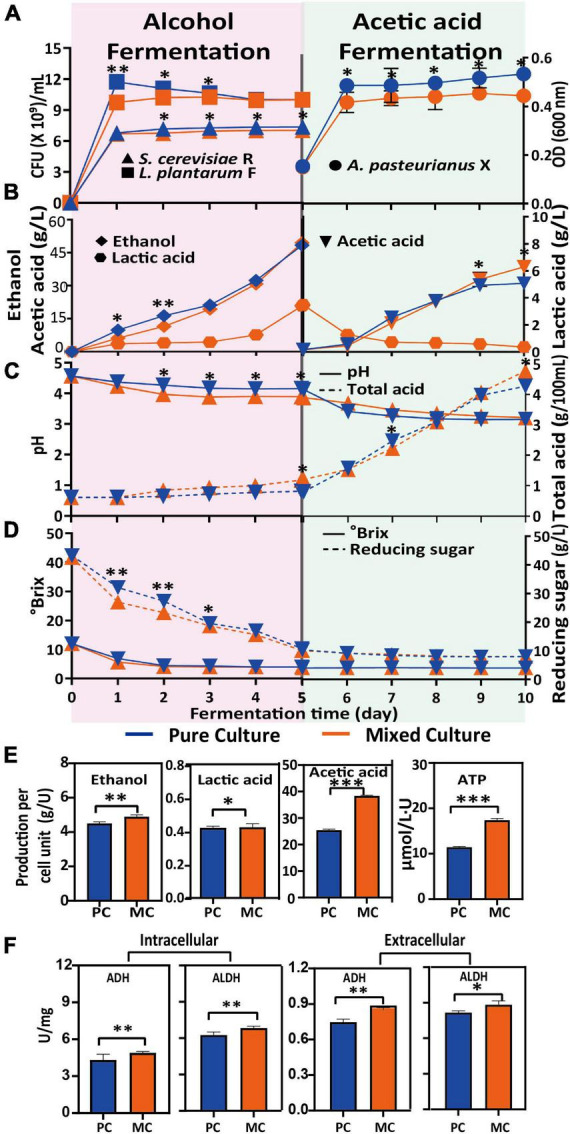
Variation in physicochemical parameters during alcoholic and acetic acid fermentation, and differences between pure culture (PC) and mixed culture (MC). Biomass increase with time of *Saccharomyces cerevisiae* R, *Lactiplantibacillus plantarum* F and *Acetobacter pasteurianus* X **(A)**; ethanol, lactic acid and acetic acid production **(B)**, pH and total acid **(C)**; Brix and reducing sugar concentration **(D)**; final production of ethanol and lactic acid after alcoholic fermentation, and acetic acid and ATP after acetic acid fermentation, per cell unit (U). One cell unit (U) is defined as 10^9^ cells (*S. cerevisiae* R *and L. plantarum* F) or cell suspension with an optical density of 1 at 600 nm wavelength (*A. pasteurianus* X) **(E)**; intracellular and extracellular ADH and ALDH activity at the end of fermentation **(F)**. From *T* test, *p < 0.05; **p < 0.01; ***p < 0.001.

A previous study (37) demonstrated that an appropriate addition of *L. plantarum* has a positive effect on alcoholic fermentation. The growth of *S. cerevisiae* R was similar under both the PC and MC conditions, and reached the highest cell density (1.3 × 10^8^ CFU/mL) on the fifth day (Figure 1A). *L. plantarum* F grew rapidly on the first day in both PC and MC, and reached its highest cell density at day one. The presence of *S. cerevisiae* R had a noticeable inhibitory effect on the growth of *L. plantarum* F, reflected as a 30% lower CFU count of *L. plantarum* F at day one in MC, compared with PC. However, by the end of the alcoholic fermentation, the counts of *L. plantarum* F were similar in both systems. There was little difference in soluble solids and total acid content between MC and PC (Figure 1C). Reducing sugar was consumed faster in MC during the first 3 days of alcoholic fermentation, but less ethanol was accumulated in MC (Figures 1B, D). These results indicated that some reducing sugar was converted to other metabolites initially. At the end of acetic acid fermentation, the alcohol content of PC and MC was 0.48 and 0.53 g/L, respectively.

By the end of acetic acid fermentation, the biomass of *A. pasteurianus* X after PC was 1.2-fold higher than that after MC, but the acetic acid content after MC was 21% higher than that after PC. At acetic acid concentrations >0.5%, the cell metabolism and growth of *A. pasteurianus* X are affected, because of decreased intracellular pH and metabolic disturbance by acetate, as well as other deleterious effects (38–41) in MC. Ethanol is oxidized to acetic acid by two sequential reactions catalyzed by ADH and ALDH, which are both located on the periplasmic side of the cytoplasmic membrane, and these reactions are linked to the respiratory chain (42). In this study, the activities of intracellular and extracellular ethanol-acetaldehyde-acetic acid respiratory chain enzymes (ADH and ALDH) in MC were significantly higher than those in PC (Figure 1F), which could explain the higher acetic acid production after MC. Moreover, this process (Ethanol is oxidized to acetic acid by two sequential reactions) generates ATP for cell metabolism, therefore, higher ATP production, but less ATP consumption owing to poorer cell growth in MC could explain the much higher ATP content detected in MC (Figure 1E).

Overall, *S. cerevisiae* R had a negative impact on *L. plantarum* F biomass, and the co-fermentation of these two strains (MC) resulted in higher acidity at the end of the alcohol fermentation, inhibited *A. pasteurianus* X growth but enhanced alcohol oxidation, consistent with the higher ADH and ALDH activity and ATP content in MC.

### 3.2. The effect of MC on total polyphenols, total flavonoids, antioxidant capacity, and water-soluble B vitamins

**FIGURE 2:**
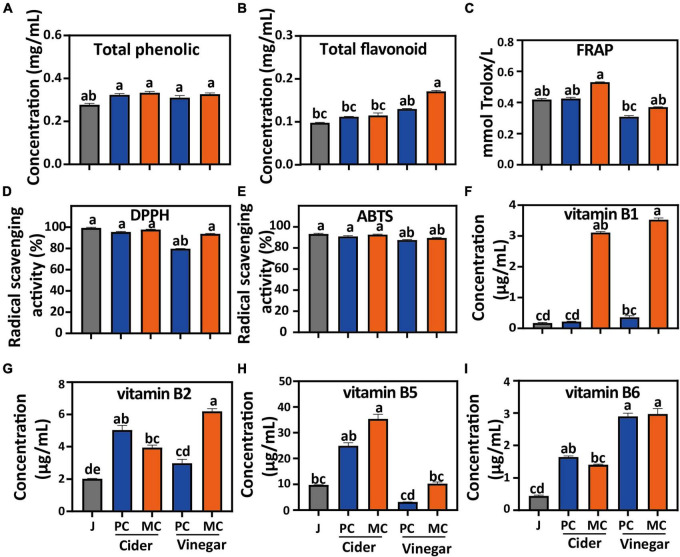
Comparison of mixed culture (MC) of *Saccharomyces cerevisiae* R and *Lactiplantibacillus plantarum* F and pure culture (PC) of *S. cerevisiae* R on: Total phenolics **(A)**; Total flavonoids **(B)**; FRAP **(C)**; DPPH **(D)** and ABTS **(E)** radical scavenging capacities; water-soluble B1 vitamins **(F)**; water-soluble B2 vitamins **(G)**; water-soluble B5 vitamins **(H)**; water-soluble B6 vitamins **(I)**. Different letters in the same figure indicate statistically significant differences in the results (*p* < 0.05).

Compared with apple juice, the total polyphenol contents of MC and PC cider increased by 16.4 and 10.7%, respectively (Figure 2A). The total flavone content of MC and PC cider increased by 21 and 20%, respectively. The total flavonoid content of MC vinegar at the end of acetic acid fermentation was 0.13 mg/mL, 1.3 times of that of PC vinegar (Figure 2B). Fermentation by *L. plantarum* increased the total polyphenol and total flavonoid contents of mulberry juice (27). The increased polyphenol content after *L. plantarum* fermentation has been attributed to its production of hydrolytic enzymes which hydrolyze complex phytochemicals, such as catechin gallate, epicatechin gallate and baicalin wogonoside into simpler forms (27, 43), which may result in higher flavonoids detected.

The DPPH and ABTS radical scavenging capacities of cider and vinegar were slightly lower than those of apple juice (Figures 2D, E). However, the DPPH radical scavenging capacity of MC cider and vinegar increased by 3 and 16%, respectively, compared with PC cider and vinegar. The ABTS radical scavenging capacity of MC cider and vinegar were slightly lower than PC cider and vinegar (*p* > 0.05). Compared with PC, the FRAP of MC cider and vinegar increased by 26 and 19%, respectively (Figure 2F). In general, MC increased the DPPH radical scavenging capacity and FRAP of cider and vinegar, which may be related to its increased flavonoid content; antioxidant capacity is closely related to polyphenol content (25).

The concentrations of water-soluble B vitamins in cider and vinegar were higher than those in apple juice (Figure 2C). In addition, the vitamin B1 and B5 contents of MC cider were 13.6- and 1.36-fold higher than those of PC cider, respectively, and the vitamin B1, B2 and B5 contents of MC vinegar were 9. 56-, 2. 09-, and 1.1-fold higher than those of PC vinegar (Figures 2G–I), respectively. The pyrimidine and thiazole ring moieties composing vitamin B1 (thiamin) are synthesized by the purine and hexose monophosphate pathways, and vitamin B2 (riboflavin) by the purine pathway, from GTP and ribulose-5-phosphate (44). Vitamin B5 is synthesized by the pantothenate and CoA biosynthesis pathway, and is related to L-valine and L-aspartate metabolism (45). To explain the increased vitamin biosynthesis in MC fermentation, comparative analysis of the metabolic pathways of MC and PC fermentation was undertaken.

### 3.3. Changes in organic acid and amino acid contents during fermentation of apple cider vinegar

**FIGURE 3:**
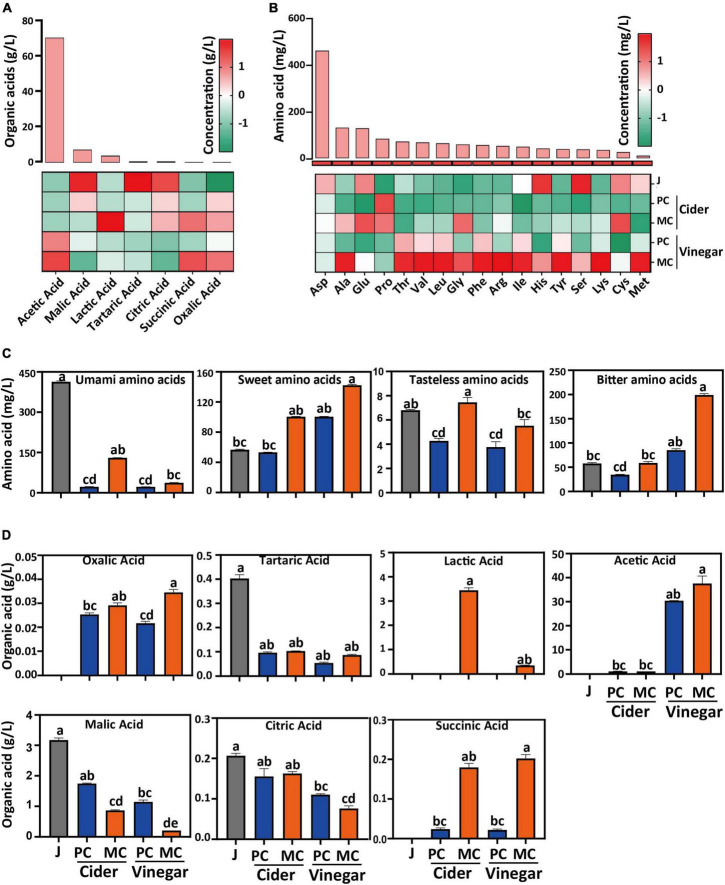
Comparison of organic acid and free amino acid content in mixed culture (MC) of *Saccharomyces cerevisiae* R and *Lactiplantibacillus plantarum* F and pure culture (PC) of *S. cerevisiae* R: Relative content **(A)** and absolute content **(D)** of organic acids; Relative content **(B)** and absolute content **(C)** of free amino acids in apple cider vinegar (relative concentration value in A and B was normalized in each column by zero-mean normalization).

Mixed culture with *L. plantarum* altered the organic acid composition of apple cider and vinegar, compared with PC (Figure 3A). Malic acid in MC decreased significantly during the alcoholic fermentation, because both *S. cerevisiae* and *L. plantarum* can metabolize malic acid as a carbon source, and *L. plantarum* can convert malic acid into lactic acid *via* malolactic fermentation. Succinic acid is an important metabolite of yeast; after alcoholic fermentation, the succinic acid concentration of MC (0.17 g/L) was 7.3-fold that of PC (0.023 g/L); after acetic acid fermentation, the succinic acid concentration of MC (0.19 g/L) was 8.3 times that of PC (0.023 g/L) (Figure 3D). Lower malic and higher succinic acid concentrations were the main differences between apple cider vinegars fermented by MC, compared with PC. The acetic acid concentration of MC was 1.3-fold that of PC after acetic acid fermentation. Lactic acid, a side product of *L. plantarum* F glycolysis, would have been used by *A. pasteurianus* X to produce acetic acid and acetoin. The other tested organic acid contents were similar between PC and MC.

Free amino acids are considered to be an important contributor to the distinctive taste of vinegar (46). After both cider and vinegar fermentations, the total free amino acid content decreased significantly compared with that in apple juice (Figure 3B). Aspartate, the major amino acid in apple juice, decreased by 97, 96, 97, and 77% in MC cider, PC cider, MC vinegar, and PC vinegar, respectively. The total free amino acids in MC cider and vinegar were 2.6- and 2.3-fold higher, respectively, than for PC cider and vinegar. Free amino acids are classified into groups with similar taste, i.e., umami, sweet, bitter, and tasteless (47, 48). Except for Asp and Pro, the free amino acid content in MC vinegar was significantly higher than that in PC vinegar (*p* < 0.05). The total umami amino acids (Asp and Glu) in MC cider and vinegar were 6.02- and 1.65-fold higher than those in PC cider and vinegar, respectively (47, 48). The total sweet amino acids (Ala, Glu, Thr, Ser, and Pro) in MC cider and vinegar were 1.88- and 2.67-fold higher than those of PC cider and vinegar, respectively (Figure 3C; 47, 48). Thr, Met, Ile, Leu, Phe, Lys, and Val are essential amino acids, and were more abundant in MC than PC. Overall, MC increased the total free amino acid content in vinegar and enhanced its taste by increasing the content of umami and sweet tasting amino acids.

### 3.4. Multivariate statistical analysis of apple cider vinegar metabolites

Base peak intensity (BPI) chromatograms in positive and negative ionization modes for PC, MC, and juice samples were recorded (Supplementary Figure 1); there were significant differences between the PC and MC vinegars. In total, 10,049 and 8,908 metabolite ion features were detected in the vinegar samples, in the positive and negative modes, respectively.

**FIGURE 4:**
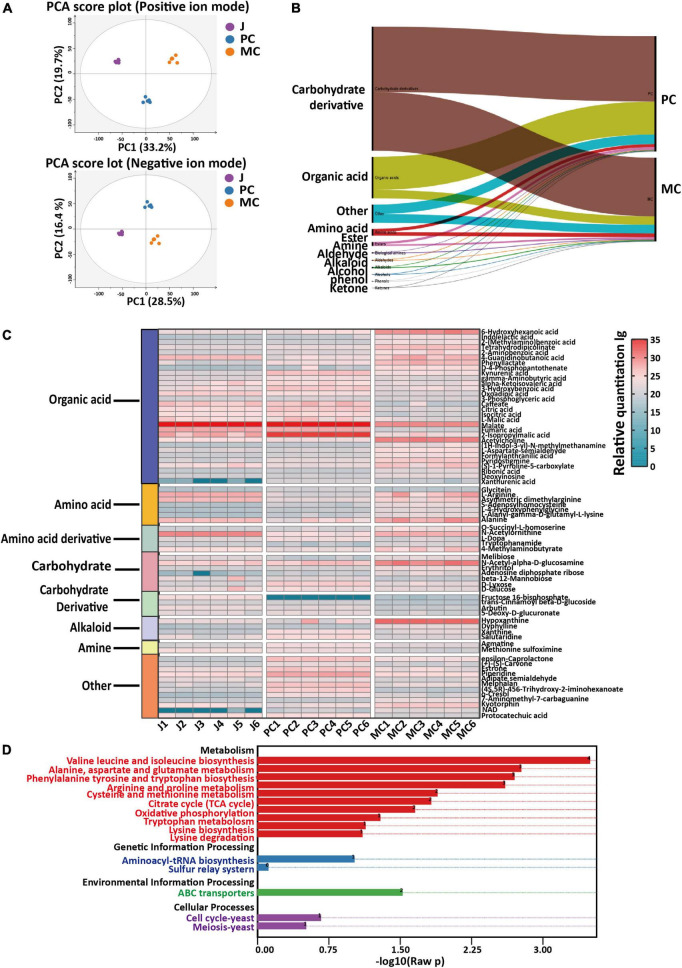
Comparison of metabolite profiles and metabolic pathways in mixed culture (MC) of *Saccharomyces cerevisiae* R and *Lactiplantibacillus plantarum* F with pure culture (PC) of *S. cerevisiae* R: PCA score plots of positive and negative ionization LC-MS data, red = J (apple juice), blue = PC and orange = MC **(A)**; classification and relative content of differential marker metabolites **(B)**; relative content of marker metabolites **(C)**; KEGG enrichment of differential metabolic pathways **(D)**.

To investigate the impact of co-fermentation on apple cider vinegar, the metabolite dataset was subjected to three-component PCA analysis (in positive and negative ion modes, Figure 4A) and the data from PC and MC samples was well separated, with significant clustering behavior (in positive ion mode: *R*^2^X = 0.529; in negative ion mode: *R*^2^X = 0.533). Four component PCA analysis included the above three sample groups as well as QC samples (mixtures of all samples) and a hierarchical cluster analysis showed similar results (Supplementary Figures 2A, B), demonstrating that the metabolic profiles of conventional PC, and MC with *L. plantarum* co-fermentation were markedly different and easily distinguished. A supervised chemometric analysis by PLS-DA (in positive and negative modes) was established using the PC and MC datasets. Distinct grouping according to fermentation process was achieved (*R*^2^X = 0.627, *R*^2^Y = 0.999, *Q*^2^ = 0.988) and 273 metabolites (with VIP > 1, *p* < 0.01) were identified as the differential compounds that differentiated PC and MC samples.

**FIGURE 5:**
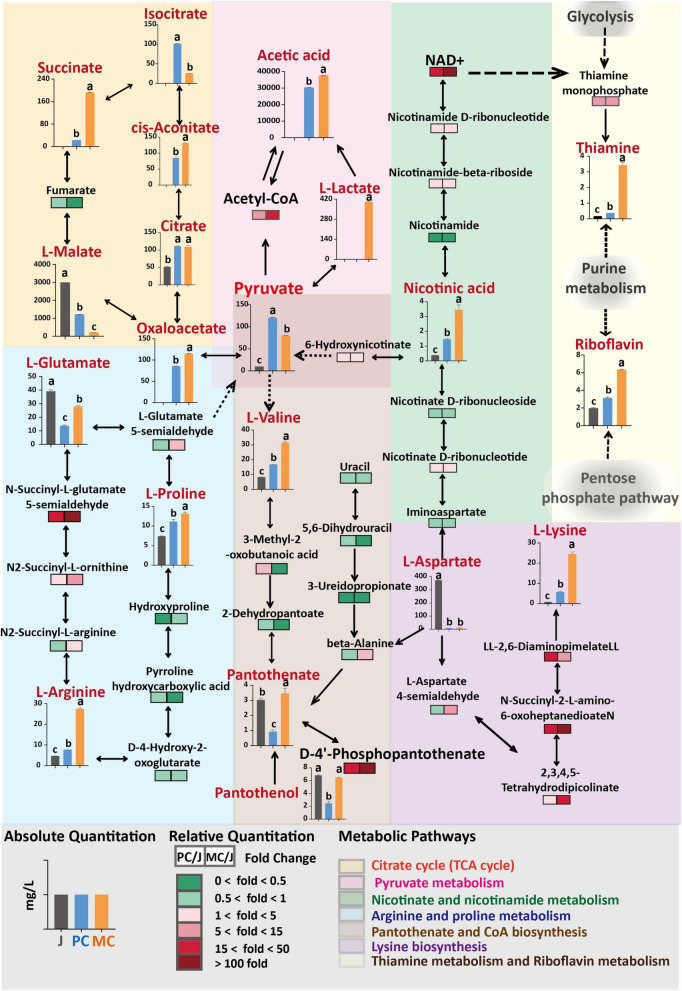
Comparison of differential metabolites and metabolic pathways in mixed culture (MC) of *Saccharomyces cerevisiae* R and *Lactiplantibacillus plantarum* F and pure culture of *S. cerevisiae* R (PC). The fold change of relative content of each metabolite compared with control (apple juice) was visualized as a heat map and combined with the possible metabolic pathways. The absolute quantity of several metabolites is visualized with charts and located in the metabolic pathways. Different letters in the same figure indicate statistically significant differences in the results (*p* < 0.05).

The relative contents of amino acids, alkaloids, amines and other metabolites in MC were higher than those in PC (Figure 4B). Based on the fold change of relative concentration (fold change ≥ 5 or ≤ 0.25; fold change = MC/PC), 71 important differential compounds were identified as candidate markers characteristic of MC and PC samples (Figure 4C; 49). Most of the markers were organic acids, or amino acids and the less abundant compound classes included alkaloids, amino acid derivatives, carbohydrates, carbohydrates derivatives and amines (Supplementary Table 1). The relative abundance of marker compounds in each sample is shown in a heatmap (Figure 5).

The relative content of amino acid, amine and alkaloid metabolites identified in MC vinegar were 1. 25-, 2. 00-, and 1.59-fold higher than those in PC vinegar, respectively. In the comparison between PC and MC apple vinegar, the relative contents of the metabolites L-arginine, acetylcholine, hypoxanthine, 6-hydroxyhexanoic acid, dyphylline, indoleacetic acid, indolelactic acid, 4-guanidinobutanoic acid, melibiose, phenyllactic acid and alanine in MC vinegar were markedly higher than those in PC, with a fold change >56.6. However, the relative contents of malic and citric acids were much lower in MC vinegar, consistent with the quantitative analysis (Section “3.3. Changes in organic acid and amino acid contents during fermentation of apple cider vinegar”).

Enrichment of differential metabolic pathways between MC and PC is shown in Figure 4D. The main metabolic pathways enriched were arginine and proline metabolism, citrate cycle (TCA cycle), lysine biosynthesis, cysteine and methionine metabolism, aspartate and glutamate metabolism and pyruvate metabolism.

### 3.5. Differential key metabolic pathways

The KEGG predictions indicated that pathways related to aspartate, pyruvate, glutamate, lysine and organic acids were significantly influenced by the fermentation process (PC and MC) (Figure 4D). The differential metabolic pathways were closely related to amino acid and organic acid metabolism, indicating that the TCA cycle, glycolysis and amino acid metabolism were active pathways during vinegar production. To elucidate the specific changes metabolites related to these metabolic pathways, relative quantification of metabolites *via* LC-MS based metabolomics, combined with absolute quantification of amino acids, organic acids, vitamin B1, B2, and B5 metabolites by HPLC was performed (Figure 5). The TCA cycle links with various catabolic and anabolic biochemical pathways, regulating the electron transport chain and finally generating ATP (50, 51). Malic acid, the major organic acid, decreased by 61% and 93% in the PC and MC vinegars, respectively, whereas citric acid increased about 2.2-fold in the PC and MC vinegars compared with apple juice. Comparing the PC and MC vinegars, many TCA cycle intermediates varied markedly. Specifically, oxaloacetic, succinic and aconitic acids were much more abundant in MC, whereas isocitric and malic acids were more abundant in PC (Figure 5). These variations suggested that in the co-fermentation with *L. plantarum* F, which has an incomplete TCA cycle, the utilization of the abundant malic acid as carbon source was enhanced. Regarding pyruvate metabolism, significantly more lactic acid, acetyl-CoA and acetic acid accumulated during MC, and correspondingly, their common precursor, pyruvate was markedly depleted during MC. The TCA cycle are important energy-yielding pathways, which generate ATP by substrate-level phosphorylation. Comparing with single strain fermentation, co-fermentation enhanced malic acid utilization and pyruvate acid metabolism were consistent with the higher ATP content detected in MC system (Figure 1E), and higher energy is beneficial for various cellar processes, and may explain the higher alcohol and acetic acid production (50, 52). The relative content of pantothenate and pantothenol in MC was higher than those in PC, and can be generated from valine or aspartate. In nicotinate and nicotinamide metabolism, NAD + is an important coenzyme for production of nicotinic acid (53), Greater accumulation of NAD + and nicotinamide in MC might explain the higher nicotinic acid production in PC. Besides, NAD + is the required cofactor for thiamin synthesis, might associated with the higher abundance of thiamin (44).

In summary, these differences in metabolic pathways between MC and PC showed that flux of malic acid metabolism in the TCA cycle and pyruvate metabolism was enhanced and energy production was higher in MC. A higher relative content of NAD + was detected in MC, which may be associated with the greater accumulation of thiamine and nicotinic acid. It appears that apple cider vinegar produced by MC may have enhanced health benefits, for example, nicotinic acid reduces progression of atherosclerosis, as well as clinical events and mortality from coronary heart disease (54).

### 3.6. Comparative analysis of volatile flavor compounds

**FIGURE 6:**
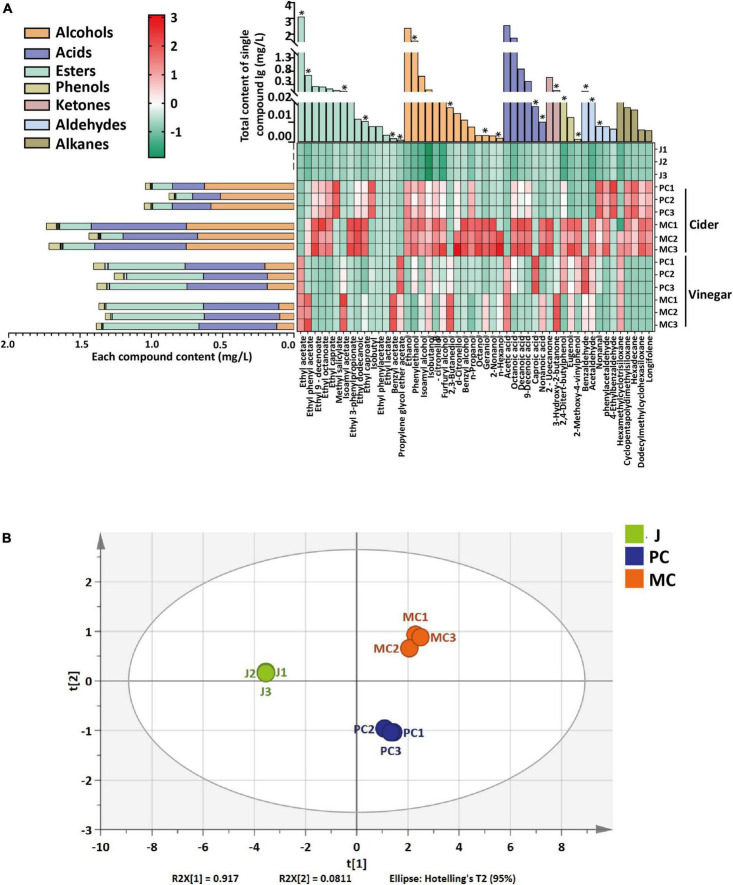
The comparative content of volatile compounds in juice, cider and vinegar prepared by PC and MC fermentation. The heatmap data was normalized within each column by zero-mean normalization **(A)**. Principal components analysis score plot of vinegar samples based on the relative abundance of volatile compounds **(B)**. The differential volatile compounds between vinegar of MC and PC systems with VIP greater than 1 are labeled with * in panel **(A)**.

Volatile compounds, including alcohols, acids, esters, ketones, phenols, aldehydes and alkenes, in cider and vinegar made by PC and MC were compared. PC and MC cider yielded 35 and 41 volatile compounds, respectively, PC and MC vinegar yielded 31 and 37 volatile compounds, respectively (Supplementary Table 2). After the alcohol fermentation, MC cider had more variety and higher content of volatile alcohols than PC cider (Figure 6A). Specifically, the contents of phenylethanol, benzyl alcohol, geraniol and n-hexanol were relatively much higher in MC cider. Geraniol and phenylethanol have rosy aromas, and n-hexanol and benzyl alcohol contribute to a fruity aroma. Volatile acids were more abundant in MC cider compared with PC cider, and octanoic acid and decanoic acid were the two major acids, which may contribute to a richer aroma and longer-lasting tastes for MC cider. The volatile ester concentration of MC vinegar was 20% higher, which is consistent with a previous report that the addition of *L. plantarum* F to the fermentation culture increased the production of esters in cherry wines (55). Ethyl acetate was the most abundant ester with a low odor threshold; the relative content of ethyl acetate in MC vinegar was slightly higher (3.85%) than that of PC vinegar (*p* < 0.05). Ethyl phenyl acetate and isoamyl acetate in MC vinegar were 5.65- and 3.47-fold more abundant than in PC vinegar (*p* < 0.05); they have a sweet and a banana-like aroma, respectively. In addition, some esters were found in MC vinegar but not in PC vinegar, such as ethyl lactate, ethyl caprate, methyl salicylate and ethyl caproate. Ethyl lactate, ethyl caprate and ethyl caproate contribute rum-like, creamy and fruity aromas. Alcohols were less abundant in MC vinegar; phenylethanol, β-citronellol and n-propanol were significantly less abundant in MC vinegar compared with PC. A PCA score plot (Figure 6B) clearly distinguished PC and MC vinegar. Overall, the mixed fermentation with *L. plantarum* F markedly modified the aroma profile of apple cider vinegar by lowering its volatile alcohol content and increasing its ester content, thereby enhancing its fruity aroma.

### 3.7. Sensory evaluation

**FIGURE 7:**
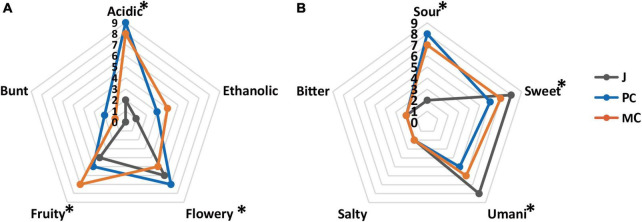
Sensory evaluation of aroma **(A)** and taste **(B)** in mixed culture vinegar and pure culture vinegar (* represents *p* < 0.05).

The sensory analysis of MC and PC vinegars is shown in Figures 7A, B (aroma) (taste). The taste of vinegar is generally considered to be dominated by sourness, followed by sweet and umami tastes, with slight salty and bitter tastes, which appears to result from interactions among the different taste components (46). MC had a significantly higher fruity aroma intensity than PC (*p* < 0.05), probably resulting from the high content of ethyl phenylacetate, isoamyl acetate and ethyl acetate in MC, whereas PC had a significantly higher flowery aroma intensity (*p* < 0.05). MC had significantly higher umami and sweet taste intensities than PC (p < 0.05), which is consistent with the significantly higher sweet and umami amino acid content of MC vinegar (*p* < 0.05). Although the total acidity of MC was higher than that of PC, MC had a significantly lower sour taste intensity (*p* < 0.05). Higher abundance of sweet and umami amino acids, and flavor esters could have masked the sour taste and sharp acidic odor of acetic acid (Figure 4D). Overall, the PC apple cider vinegar had a stronger flowery aroma, whereas the MC vinegar had a stronger fruity aroma and stronger sweet and umami tastes.

## 4. Conclusion

This study demonstrated that alcoholic fermentation of apple juice using a mixed culture of *S. cerevisiae* R and *L. plantarum* F can significantly improve the flavor and quality of the final vinegar. LC-MS based metabolomics analysis was applied to compositional variations between PC and MC vinegars, and combined with quantification of organic acids, amino acids and B vitamins. A total of 71 differential metabolites including amino acids, organic acids and carbohydrates were identified, and six possible key metabolic pathways which could account for the differential metabolites were elucidated. MC appeared to enhance the malic acid utilization and pyruvate acid metabolism, thereby increasing substrate level phosphorylation, and yielding more energy for cellular metabolism. Higher acidity resulting from lactic acid accumulation at the beginning of the acetic acid fermentation after MC fermentation resulted in greater suppression of cellular metabolism and cell growth of *A. pasteurianus*, but enhanced its ethanol-acetaldehyde-acetic acid respiratory chain, which stimulated ATP accumulation, and higher acetic acid production. MC vinegar had higher contents of B vitamins, pantothenate, total flavonoids, total organic acids, amino acids and higher antioxidant capacity. MC fermentation enhanced the volatile content of the resulting apple cider vinegar, particularly ethyl lactate, ethyl caprate and ethyl caproate, which contribute to a fruity aroma. This is consistent with the sensory evaluation, i.e., MC apple cider vinegar had a higher fruity aroma intensity, as well as stronger umami and sweet tastes. These findings improve understanding of the metabolic mechanism of multi-strain collaborative vinegar fermentation processes. Future studies should focus on using mixed strains to rationally modify the flavor and increase the functional substance content of apple cider vinegar.

## Data availability statement

The original contributions presented in this study are included in the article/Supplementary material, further inquiries can be directed to the corresponding authors.

## Author contributions

Y-NL: conceptualization, data curation, methodology, and writing–original draft. X-JZ and Z-HX: conceptualization, funding acquisition, supervision, and writing–review and editing. YL: investigation. Z-ML: data curation and methodology. L-JC: resources. Y-LD: validation. J-SS: supervision. All authors contributed to the article and approved the submitted version.

## Footnotes

1
http://www.hmdb.ca/

2
https://metlin.scripps.edu/
