# Supplementary material for: Metabolomic analysis of the effects of a mixed culture of Saccharomyces cerevisiae and Lactiplantibacillus plantarum on the physicochemical and quality characteristics of apple cider vinegar
Source: Front Nutr. 2023 Mar 14;10:1142517. doi: 10.3389/fnut.2023.1142517 (PMC10043408; doi:10.3389/fnut.2023.1142517)
Supplement: Supplementary file 1 [file Data_Sheet_1.doc]

Metabolomic analysis of the effects of a mixed culture of *Saccharomyces cerevisiae* and *Lactiplantibacillus plantarum* on the physicochemical and quality characteristics of apple cider vinegar

Ya-Nan Li ^a,b^, Yue Luo ^a,b^, Zhen-Ming Lu ^a,b^, Yan-Lin Dong ^d^, Li-Juan Chai ^b,e^, Jin-Song Shi ^c,e^, Xiao-Juan Zhang ^a,b*^, Zheng-Hong Xu ^a,b*^

^a^ Key Laboratory of Industrial Biotechnology of Ministry of Education, School of Biotechnology, Jiangnan University, Wuxi, China

^b^ National Engineering Research Center of Cereal Fermentation and Food Biomanufacturing, Jiangnan University, Wuxi, China

^c^ School of Life Science and Health Engineering, Jiangnan University, Wuxi, China

^d^ Apple Cider Vinegar Engineering & Technology Research Center of Yantai, Lvjie Co., Ltd, Yantai, China

^e^ Jiangsu Engineering Research Center for Bioactive Products Processing Technology, Jiangnan University, Wuxi, China

*Corresponding author at: National Engineering Research Center for Cereal Fermentation and Food Biomanufacturing, Jiangnan University, 1800 Lihu Avenue, Wuxi 214122, P.R. China

E-mail address: zhangxj@jiangnan.edu.cn(X.-J. Zhang) or zhenghxu@jiangnan.edu.cn (Z.-H. Xu)

Note 1. The detailed isolation procedure screening strain.

*L. plantarum* F: The vinegar pei sample of cereal vinegar fermented at 7th day of alcohol fermentation was weighed to 1g, then added to 100ml sterile water and incubated at 37℃ for 1d. Next, the supernatant was coated on MRS solid medium (Peptone 10 g, beef extract 10 g, yeast extract 5 g, diammonium citrate 2 g, sodium acetate 5 g, K_2_HPO_4_ 2 g, MnSO_4_ • 4H_2_O 0.25 g, MgSO_4_ • 7H_2_O 0.58 g, glucose 20 g, tween 80 mL, agar 25 g, water 1000 mL) after gradient dilution, and incubated at 37℃ for 1 d. The DNA of a single colony was extracted using the bacterial genomic DNA rapid extraction kit (Shanghai, Sangong Bioengineering Co., LTD.). For specific operation steps, refer to the product manual. Total bacterial DNA was used as a template using primer 27F (5'-AGAGTTTGATCCTGGCTCAG-3') and 1492R (5'-TACCTTGTTACGACTT-3') were amplified and sequenced for the 16S rRNA gene.

*A. pasteurianus* X: The vinegar pei sample of cereal vinegar fermented at 18th day of acetic acid fermentation was weighed to 1g, then added to 100ml sterile water and incubated at 30℃ for 2d. Next, the supernatant was coated on LB solid medium (yeast extract 10 g, glucose 20 g, agar 25 g, ethanol 2%, water 1000 mL) after gradient dilution, and incubated at 30℃ for 2 d. The DNA of a single colony was extracted using the bacterial genomic DNA rapid extraction kit (Shanghai, Sangong Bioengineering Co., LTD.). For specific operation steps, refer to the product manual. Total bacterial DNA was used as a template using primer 27F (5'-AGAGTTTGATCCTGGCTCAG-3') and 1492R (5'-TACCTTGTTACGACTT-3') were amplified and sequenced for the 16S rRNA gene.

*S. cerevisiae* R: The vinegar pei sample of cereal vinegar fermented at 7th day of alcohol fermentation was weighed to 1g, then added to 100ml sterile water and incubated at 37℃ for 1d. Next, the supernatant was coated on YPD solid medium (yeast extract 10 g, glucose 20 g, peptone 20 g, agar 25 g, water 1000 mL) after gradient dilution, and incubated at 37℃ for 1 d. The DNA of a single colony was extracted using the bacterial genomic DNA rapid extraction kit (Shanghai, Sangong Bioengineering Co., LTD.). For specific operation steps, refer to the product manual. Total bacterial DNA was used as a template using primer ITS1F (5'-GTCGTAACAAGGTTTCCGTAGGTG-3') and ITS1R (5'-TCCTCCGCTTATTGAGATGC-3') were amplified and sequenced for the 16S rRNA gene.

Table S1

The marker metabolites of apple cider vinegar for distinguishing Pure culture and Mixed culture.

| NO. | Var ID | Rt (s) | m/z | MS | MS/MS | Exact mass | Mass error (ppm) | Identification | formula | VIP | Classification |
| --- | --- | --- | --- | --- | --- | --- | --- | --- | --- | --- | --- |
| 1 | M116T76 | 76.49 | 116.01 | [M]- | 115.003, 73.0296, 72.0166, 71.0133 | 116.011 | 8.95 | Fumaric acid | C4H4O4 | 1.86 | Organic acid |
| 2 | M133T76_3 | 76.05 | 133.01 | [M-H]- | 133.0143, 115.0035, 72.9931, 71.0139 | 134.02 | 7.14 | Malate | C4H6O5 | 1.85 | Organic acid |
| 3 | M135T155 | 155.23 | 135.03 | [M-H]- | 135.0305, 92.025 | 136.03 | 6.68 | Hypoxanthine | C5H4N4O | 1.85 | Alkaloids |
| 4 | M107T305 | 305.40 | 107.05 | [M-H]- | 108.0453, 107.0503, 106.042, 65.0143 | 108.05 | 0.62 | o-Cresol | C7H8O | 1.84 | other |
| 5 | M116T92 | 91.64 | 116.04 | [M-H]- | 116.9289, 116.0715, 104.9867, 103.9898, 102.9885, 99.9262, | 117.04 | 0.93 | L-Aspartate-semialdehyde | C4H7NO3 | 1.84 | other |
| 6 | M339T71 | 70.83 | 338.99 | [M-H]- | 338.9875, 320.9799, 241.0119, 158.9252, 96.9695, 78.9592 | 339.99 | 2.72 | Fructose 1,6-bisphosphate | C6H14O12P2 | 1.84 | Carbohydrates |
| 7 | M283T315 | 314.56 | 283.07 | [M-H]- | 283.1635, 70.3842 | 284.06 | 25.63 | Glycitein | C16H12O5 | 1.82 | other |
| 8 | M204T326 | 326.29 | 204.03 | [M-H]- | 204.0293, 161.0428, 160.0394, 69.6155 | 205.03 | 0.07 | Xanthurenic acid | C10H7NO4 | 1.82 | other |
| 9 | M153T120 | 120.40 | 153.02 | [M-H]- | 154.0412, 153.0191, 135.0456, 125.0251, 110.0281, 109.0292 | 154.02 | 0.10 | Protocatechuic acid | C7H6O4 | 1.80 | Other |
| 10 | M169T293 | 293.04 | 169.01 | [M-H]- | 169.0141, 151.0046, 125.0241, 108.0453, 68.8511 | 170.02 | 0.02 | Gallic acid | C7H6O5 | 1.80 | Organic acid |
| 11 | M137T149 | 148.54 | 137.02 | [M-H]- | 137.0233, 109.0298, 94.0367, 93.034 | 138.03 | 4.51 | 3-Hydroxybenzoic acid | C7H6O3 | 1.78 | Organic acid |
| 12 | M167T345 | 344.82 | 167.06 | [M]- | 167.0568, 166.055, 149.0506, 148.048, 147.0451, 120.0492, | 167.06 | 13.27 | L-4-Hydroxyphenylglycine | C8H9NO3 | 1.78 | Amino acid |
| 13 | M309T314 | 314.46 | 309.10 | [M-H]- | 309.0931, 237.9473, 181.0709, 137.0606, 129.0187, 127.004, | 310.11 | 0.69 | trans-Cinnamoyl beta-D-glucoside | C15H18O7 | 1.78 | Carbohydrates derivative |
| 14 | M166T415 | 414.73 | 166.05 | [M]- | 167.0339, 166.051, 165.0778, 147.0455, 122.0611, 103.0401, | 166.05 | 14.16 | Ribonic acid | C5H10O6 | 1.77 | other |
| 15 | M175T137_1 | 137.35 | 175.06 | [M-H]- | 175.0619, 157.051, 131.0721, 115.0408, 113.0611, 85.0666 | 176.07 | 8.82 | 2-Isopropylmalic acid | C7H12O5 | 1.76 | Organic acid |
| 16 | M342T165 | 165.25 | 342.12 | [M]- | 342.1164, 222.0747, 180.0674, 179.0453, 163.0409, 135.0454, | 342.17 | 8.68 | Melibiose | C12H22O11 | 1.73 | Carbohydrates |
| 17 | M177T137 | 137.19 | 177.07 | [M]- | 178.0513, 177.0477, 176.0497, 159.0295, 148.9611, 147.9646, | 177.06 | 9.81 | (4S,5R)-4,5,6-Trihydroxy-2-iminohexanoate | C6H11NO5 | 1.73 | other |
| 18 | M147T345 | 344.87 | 147.05 | [M-H2O-H]- | 147.0452, 119.05, 103.055, 101.04, 61.9886 | 166.06 | 3.36 | Phenyllactate | C9H10O3 | 1.70 | Organic acid |
| 19 | M203T365 | 364.73 | 203.11 | [M]- | 204.0669, 203.0839, 202.114, 186.0564, 159.0921, 142.0667, | 203.11 | 29.65 | Tryptophanamide | C11H13N3O | 1.69 | Amino acid derivative |
| 20 | M131T237 | 236.88 | 131.07 | [M-H]- | 131.0711, 85.0655 | 132.08 | 9.63 | 6-Hydroxyhexanoic acid | C6H12O3 | 1.68 | Organic acid |
| 21 | M179T224 | 223.73 | 179.03 | [M-H]- | 179.0352, 136.0476, 135.0449 | 180.04 | 1.85 | Caffeate | C9H8O4 | 1.64 | Organic acid |
| 22 | M181T208_2 | 208.15 | 181.06 | [M+H]+ | 181.9699, 181.062, 180.0647, 176.9839, 163.0497, 158.9725, | 180.06 | 18.09 | Methionine sulfoximine | C5H12N2O3S | 1.58 | Amine |
| 23 | M181T335 | 334.86 | 181.10 | [M+H]+ | 182.1008, 181.0972, 125.071 | 181.09 | 0.051 | Pyridostigmine | C9H13N2O2 | 1.57 | other |
| 24 | M122T360 | 359.71 | 122.06 | [M]+ | 123.0497, 122.0602, 116.9662, 112.0391, 105.0439, 104.0497, | 122.05 | 18.53 | Erythritol | C4H10O4 | 1.57 | Carbohydrates |
| 25 | M305T163 | 162.70 | 305.08 | [M+H]+ | 305.0834, 288.0564, 126.092, 124.0399, 84.0804 | 304.07 | 0.02 | Melphalan | C13H18Cl2N2O2 | 1.57 | other |
| 26 | M193T151 | 150.51 | 193.03 | [M+H]+ | 194.1171, 192.0659, 164.0708, 157.0137, 139.002, 135.0442, | 192.03 | 0.49 | Isocitric acid | C6H8O7 | 1.57 | Organic acid |
| 27 | M175T150 | 150.49 | 175.02 | [M+H-H2O]+ | 194.1171, 192.0659, 164.0708, 157.0137, 139.002, 135.0442, | 192.03 | 22.05 | Citric acid | C6H8O7 | 1.56 | Organic acid |
| 28 | M86T107 | 106.90 | 86.10 | [M+H]+ | 86.1481, 69.0849 | 85.09 | 0.18 | Piperidine | C5H11N | 1.56 | other |
| 29 | M253T340 | 340.32 | 253.09 | [M+H]+ | 254.1604, 253.0967, 226.8936, 195.1137, 137.0457, 136.062, | 252.08 | 0.32 | Deoxyinosine | C10H12N4O4 | 1.56 | Other |
| 30 | M328T163 | 162.70 | 328.16 | [M+H]+ | 329.0861, 328.1426, 311.1261, 293.1113, 275.0745, 263.1046, | 327.15 | 16.18 | Salutaridine | C19H21NO4 | 1.56 | Alkaloids |
| 31 | M175T101 | 100.90 | 175.11 | [M+H]+ | 175.1072, 129.1022, 116.0704, 112.0755, 70.0655 | 174.10 | 0.01 | N-Acetylornithine | C7H14N2O3 | 1.56 | Amino acid |
| 32 | M162T288 | 288.43 | 162.08 | [M+H-H2O]+ | 163.0388, 162.078, 161.06, 151.0375, 145.0073, 144.0668, | 179.08 | 19.91 | 7-Aminomethyl-7-carbaguanine | C7H9N5O | 1.56 | Other |
| 33 | M172T175 | 175.09 | 172.06 | [M+H]+ | 172.0868, 171.1492, 166.9878, 154.0863, 149.9876, 148.9892, | 171.05 | 0.04 | Tetrahydrodipicolinate | C7H9NO4 | 1.56 | Organic acid |
| 34 | M153T318 | 317.55 | 153.04 | [M+H]+ | 154.0246, 153.0408, 152.0567, 136.014, 110.035 | 152.03 | 0.62 | Xanthine | C5H4N4O2 | 1.56 | Alkaloids |
| 35 | M150T101 | 100.79 | 150.08 | [M]+ | 151.072, 150.0776 | 150.05 | 0.81 | D-Lyxose | C5H10O5 | 1.55 | Carbohydrates |
| 36 | M130T557 | 557.33 | 130.12 | [M]+ | 148.9778, 138.0339, 131.0478, 130.105, 125.9605, 120.0233, | 130.12 | 6.61 | Agmatine | C5H14N4 | 1.55 | Amine |
| 37 | M190T416 | 415.80 | 190.05 | [M+H]+ | 191.0531, 190.0499, 162.0546 | 189.04 | 0.02 | Kynurenic acid | C10H7NO3 | 1.54 | Organic acid |
| 38 | M337T83 | 82.81 | 337.17 | [M]+ | 337.17, 319.1595, 260.1128, 257.1602, 242.1034, 232.1162, | 337.17 | 3.81 | Kyotorphin | C15H23N5O4 | 1.54 | Other |
| 39 | M146T101_2 | 101.31 | 146.12 | [M+H]+ | 146.1173, 100.0752, 87.0445, 69.0334, 60.0814 | 146.12 | 4.65 | Acetylcholine | C7H16NO2 | 1.54 | other |
| 40 | M115T455 | 455.36 | 115.07 | [M+H]+ | 115.9636, 115.0671, 114.0596, 92.9483, 90.9751, 87.0443, | 114.07 | 4.29 | epsilon-Caprolactone | C6H10O2 | 1.54 | other |
| 41 | M104T350 | 349.59 | 104.11 | [M+H]+ | 104.089, 87.0627, 86.0784, 69.0519, 62.9294, 60.0811, | 103.06 | 5.81 | gamma-Aminobutyric acid | C4H9NO2 | 1.54 | Organic acid |
| 42 | M254T598 | 597.66 | 254.10 | [M]+ | 181.0709, 180.0665 | 254.10 | 1.15 | Dyphylline | C10H14N4O4 | 1.54 | Alkaloids |
| 43 | M206T547 | 547.38 | 206.08 | [M+H]+ | 207.0837, 206.0809, 188.0702, 170.0594, 161.0792, 160.0753, | 205.07 | 0.15 | Indolelactic acid | C11H11NO3 | 1.53 | Organic acid |
| 44 | M161T119 | 119.40 | 161.04 | [M+H]+ | 162.0765, 161.0964, 160.0907, 151.9768, 144.0655, 143.0347, | 160.03 | 0.21 | Oxoadipic acid | C6H8O5 | 1.52 | Organic acid |
| 45 | M166T378 | 378.05 | 166.05 | [M+H]+ | 166.0587, 165.1386, 138.0552, 120.0714, 108.0446 | 165.04 | 5.27 | Formylanthranilic acid | C8H7NO3 | 1.51 | other |
| 46 | M151T534 | 534.35 | 151.11 | [M+H]+ | 152.0159, 151.1116, 123.0801, 109.0649, 105.0699, 81.0699 | 150.10 | 5.14 | (+)-(S)-Carvone | C10H14O | 1.51 | other |
| 47 | M385T175 | 174.96 | 385.13 | [M+H]+ | 136.0622, 134.0276, 88.0218 | 384.12 | 1.48 | S-Adenosylhomocysteine | C14H20N6O5S | 1.50 | Amino acid |
| 48 | M270T137 | 136.57 | 270.15 | [M]+ | 271.1279, 270.0718, 269.1479, 253.1169, 252.0862, 193.0975, | 270.16 | 1.78 | Estrone | C18H22O2 | 1.49 | other |
| 49 | M346T346 | 346.38 | 346.20 | [M]+ | 347.1127, 293.1493, 233.1121, 204.1223, 186.1136, 185.1221, | 346.18 | 1.49 | L-Alanyl-gamma-D-glutamyl-L-lysine | C14H26N4O6 | 1.49 | Amino acid |
| 50 | M152T431 | 430.62 | 152.07 | [M+H]+ | 152.0708, 151.0621, 134.0592, 108.0448, 106.0645, 80.0493, | 151.06 | 4.01 | 2-(Methylamino)benzoic acid | C8H9NO2 | 1.48 | Organic acid |
| 51 | M198T196 | 196.45 | 198.08 | [M+H]+ | 217.0295, 199.0177, 198.0757, 192.9807, 176.9853, 174.9684, | 197.06 | 5.30 | L-Dopa | C9H11NO4 | 1.47 | Amino acid |
| 52 | M325T169 | 168.82 | 325.11 | [M+H-H2O]+ | 326.0832, 325.1025, 307.0902, 255.0145, 234.9872, 199.9771, | 342.11 | 13.48 | beta-1,2-Mannobiose | C12H22O11 | 1.45 | Carbohydrates |
| 53 | M116T114 | 113.66 | 116.07 | [M-H]- | 116.9289, 115.9963, 104.9866, 103.9898, 102.9886, 99.9263, | 117.07 | 0.82 | 4-Methylaminobutyrate | C5H11NO2 | 1.45 | Amino acid derivative |
| 54 | M662T149 | 148.69 | 662.09 | [M-H]- | 540.055, 426.0202, 408.0126, 346.0558, 328.0429, 272.9564, | 664.11 | 10.42 | NAD | C21H28N7O14P2 | 1.44 | Other |
| 55 | M131T771 | 771.31 | 131.07 | [M+H]+ | 149.9413, 148.9777, 131.9752, 130.9982, 130.1318, 125.9604, 113.9638, 112.9616, 108.914, 107.9868, 97.0075, 90.9029, 89.9978, | 130.06 | 4.03 | Adipate semialdehyde | C6H10O3 | 1.41 | other |
| 56 | M540T149 | 148.97 | 540.05 | [M-H2O-H]- | 540.0605, 426.0196, 408.0138, 346.0586, 328.0428, 272.9584, | 559.07 | 4.66 | Adenosine diphosphate ribose | C15H23N5O14P2 | 1.41 | Carbohydrates |
| 57 | M299T144 | 143.67 | 299.10 | [M-H2O+NH4]+ | 299.0987, 137.0564, 97.0286 | 299.07 | 1.25 | D-4-Phosphopantothenate | C9H18NO8P | 1.38 | Organic acid |
| 58 | M273T310 | 309.75 | 273.10 | [M+H]+ | 291.5658, 273.0948, 224.8971, 208.8816, 206.8888, 175.0605, | 272.08 | 1.34 | Arbutin | C12H16O7 | 1.38 | Carbohydrates derivative |
| 59 | M117T369 | 368.93 | 117.05 | [M+H]+ | 118.0865, 76.933, 59.0734, 58.0655 | 116.04 | 0.10 | alpha-Ketoisovaleric acid | C5H8O3 | 1.36 | Organic acid |
| 60 | M222T91_2 | 91.05 | 222.10 | [M+H]+ | 223.0595, 222.0205, 205.0525, 186.0751, 176.0932, 165.056, | 221.08 | 0.75 | N-Acetyl-alpha-D-glucosamine | C8H15NO6 | 1.34 | Carbohydrates |
| 61 | M114T119 | 118.90 | 114.06 | [M+H]+ | 115.0575, 114.041, 113.0287, 97.0329, 96.0262, 95.0309, | 113.04 | 0.46 | (S)-1-Pyrroline-5-carboxylate | C5H7NO2 | 1.32 | other |
| 62 | M203T94_2 | 93.52 | 203.15 | [M+H]+ | 204.071, 203.1256, 186.0752, 168.0654, 158.1039, 144.0656, | 202.14 | 0.29 | Asymmetric dimethylarginine | C8H18N4O2 | 1.31 | Amino acid |
| 63 | M137T315 | 315.41 | 137.05 | [M]+ | 146.9618, 138.0557, 136.9994, 136.0417, 128.9503, 122.0359, 118.9893, | 137.04 | 9.22 | 2-Aminobenzoic acid | C7H7NO2 | 1.28 | Organic acid |
| 64 | M146T102 | 102.25 | 146.09 | [M+H]+ | 146.1046, 100.0752, 87.0441, 86.0598, 85.0287, 82.0648, | 145.08 | 0.13 | 4-Guanidinobutanoic acid | C5H11N3O2 | 1.25 | Organic acid |
| 65 | M202T142 | 142.47 | 202.07 | [M+H-H2O]+ | 203.183, 202.0942, 102.1005, 62.0602 | 219.07 | 20.74 | O-Succinyl-L-homoserine | C8H13NO6 | 1.25 | Amino acid |
| 66 | M90T86 | 85.84 | 90.05 | [M+H]+ | 108.9582, 98.5119, 90.9475, 90.0732, 88.0041, 87.0043, | 89.04 | 0.74 | Alanine | C3H7NO2 | 1.22 | Amino acid |
| 67 | M180T102 | 101.67 | 180.06 | [M]+ | 181.0972, 180.0659, 145.0491, 137.0714, 136.0757, 135.0441, | 180.06 | 27.47 | D-Glucose | C6H12O6 | 1.19 | Carbohydrates |
| 68 | M133T201 | 201.39 | 133.01 | [M-H]- | 134.0477, 133.014, 115.0031, 89.0242, 72.9929, 71.0139 | 134.02 | 3.46 | L-Malic acid | C4H6O5 | 1.11 | Organic acid |
| 69 | M175T82 | 81.63 | 175.12 | [M+H]+ | 175.1193, 158.0922, 157.1092, 130.097, 116.0703, 112.0866, | 174.11 | 0.41 | L-Arginine | C6H14N4O2 | 1.10 | Amino acid |
| 70 | M160T186 | 186.47 | 160.10 | [M]+ | 160.0748, 149.985, 142.0224, 131.9742, 129.9521, 128.9502, | 160.10 | 22.02 | (1H-Indol-3-yl)-N-methylmethanamine | C10H12N2 | 1.08 | other |
| 71 | M179T483 | 482.56 | 179.07 | [M+H]+ | 180.101, 179.0704, 178.0869, 174.9855, 162.0917, 161.0598, | 178.04 | 0.02 | 5-Deoxy-D-glucuronate | C6H10O6 | 1.07 | Carbohydrates derivative |

Table S2

Concentration of volatile compounds in juicy, cider and vinegar (mg/L).

|  | | | | Apple cider | | | | | | apple cider vinegar | | | | | |  |
| --- | --- | --- | --- | --- | --- | --- | --- | --- | --- | --- | --- | --- | --- | --- | --- | --- |
| Volatile compounds | J1 | J2 | J3 | PC1 | PC2 | PC3 | MC1 | MC2 | MC3 | PC1 | PC2 | PC3 | MC1 | MC2 | MC3 | Oder description ^a^ |
| Alcohols |  |  |  |  |  |  |  |  |  |  |  |  |  |  |  |  |
| Phenylethanol | 0.15 | 0.20 | 0.15 | 169.47 | 114.35 | 149.79 | 254.55 | 216.73 | 264.40 | 110.58 | 115.13 | 131.34 | 16.17 | 16.25 | 16.25 | Rose |
| Ethanol | ND | ND | ND | 320.97 | 318.79 | 387.44 | 377.01 | 361.04 | 377.32 | 46.72 | 37.50 | 37.76 | 50.20 | 37.82 | 37.82 | ND |
| n-Propanol | ND | ND | ND | 0.91 | 0.96 | 1.07 | 1.48 | 1.26 | 1.27 | 0.29 | 0.24 | 0.29 | ND | ND | ND | ND |
| Isobutanol | ND | ND | ND | 7.12 | 6.83 | 7.81 | 10.44 | 9.60 | 12.03 | 8.73 | 9.29 | 8.82 | 9.32 | 9.46 | 9.46 | black tea |
| Furfuryl alcohol | 0.17 | 0.14 | 0.13 | 2.16 | 1.75 | 1.79 | 3.49 | 2.75 | 4.55 | 1.54 | 1.29 | 1.54 | 2.16 | 2.17 | 2.17 | ND |
| Benzyl alcohol | ND | ND | ND | ND | ND | ND | 3.89 | 3.13 | 3.84 | ND | ND | ND | 0.15 | 0.16 | 0.16 | fruity |
| β- citronellol | ND | ND | ND | 2.86 | 2.94 | 2.86 | 5.16 | 4.16 | 5.10 | 1.06 | 1.31 | 1.60 | 0.53 | 0.55 | 0.55 | Rose |
| Geraniol | ND | ND | ND | ND | ND | ND | 0.83 | 0.67 | 0.82 | ND | ND | ND | 0.35 | 0.36 | 0.36 | Rose, sweet |
| n-Hexanol | 0.02 | 0.02 | 0.02 | ND | ND | ND | 0.73 | 0.60 | 0.74 | ND | ND | ND | ND | ND | ND | fruity and fat |
| Octanol | ND | ND | ND | ND | ND | ND | 1.06 | 1.05 | 1.29 | ND | ND | ND | ND | ND | ND | ND |
| Isoamyl alcohol | 0.99 | 0.91 | 1.00 | 77.05 | 68.84 | 75.80 | 96.20 | 68.74 | 74.84 | 17.85 | 20.56 | 20.56 | 37.73 | 29.22 | 35.06 | Apple brandy, spicy |
| 2,3-Butanediol | 0.04 | 0.04 | 0.04 | ND | ND | ND | ND | ND | ND | 1.55 | 1.30 | 1.55 | 4.18 | 4.53 | 4.35 | ND |
| 2-Nonanol | ND | ND | ND | ND | ND | ND | ND | 0.67 | 0.83 | ND | ND | ND | 0.09 | 0.09 | 0.09 | ND |
| d-Citronellol | ND | ND | ND | 1.38 | 1.15 | 1.38 | ND | 3.99 | 6.69 | ND | ND | ND | ND | ND | ND | ND |
| Acids |  |  |  |  |  |  |  |  |  |  |  |  |  |  |  |  |
| Acetic acid | 0.17 | 0.18 | 0.20 | 1.16 | 1.05 | 0.99 | ND | ND | ND | 410.28 | 321.89 | 409.02 | 483.47 | 470.10 | 470.10 | spicy |
| Nonanoic acid | 0.12 | 0.12 | 0.12 | ND | ND | ND | 2.09 | 1.78 | 2.35 | ND | ND | ND | 1.20 | 1.25 | 1.25 | ND |
| Octanoic acid | ND | ND | ND | 165.37 | 115.84 | 138.91 | 315.89 | 261.90 | 337.22 | 115.05 | 95.90 | 115.05 | 40.32 | 35.45 | 35.45 | Fruity |
| Caproic acid | ND | ND | ND | ND | ND | ND | ND | ND | ND | 6.13 | 5.11 | 6.13 | 0.25 | 0.26 | 0.26 | sweaty, rancid, sour, sharp, pungent, cheesy, fatty, |
| Decanoic acid | ND | ND | ND | 60.82 | 48.44 | 41.88 | 218.57 | 169.80 | 217.59 | 31.08 | 24.99 | 29.98 | 16.54 | 17.21 | 17.21 | ND |
| 9-Decenoic acid | ND | ND | ND | ND | ND | ND | 104.76 | 88.84 | 109.04 | ND | ND | ND | 3.91 | 4.07 | 4.07 | Fatty, fruity, milk |
| Esters |  |  |  |  |  |  |  |  |  |  |  |  |  |  |  |  |
| Propylene glycol ether acetate | ND | ND | ND | ND | ND | ND | ND | ND | ND | 0.25 | 0.20 | 0.25 | 0.11 | 0.11 | 0.11 | ND |
| Ethyl lactate | 0.02 | 0.01 | 0.01 | ND | ND | ND | ND | ND | ND | ND | ND | ND | 0.60 | 0.60 | 0.60 | Rum - like, fruity and creamy |
| Ethyl phenylacetate | 0.06 | 0.06 | 0.06 | 23.86 | 19.87 | 25.88 | 57.42 | 51.10 | 65.93 | 20.14 | 20.94 | 20.94 | 117.54 | 97.97 | 135.45 | Rose, sweet, honey |
| Ethyl octanoate | ND | ND | ND | 27.41 | 22.64 | 29.90 | 61.07 | 35.71 | 42.73 | 0.23 | 0.19 | 0.23 | 0.06 | 0.08 | 0.08 | Pineapple, sweet |
| Ethyl dodecanoic | ND | ND | ND | 0.91 | 0.92 | 1.20 | 2.22 | 3.31 | 3.25 | ND | ND | ND | ND | ND | ND | ND |
| Ethyl caprate | ND | ND | ND | 21.81 | 21.18 | 23.59 | 28.60 | 22.27 | 28.90 | ND | ND | ND | 1.99 | 1.66 | 1.86 | Coconut |
| Methyl salicylate | ND | ND | ND | 28.72 | 24.93 | 27.43 | 0.70 | 0.47 | 0.70 | ND | ND | ND | 0.07 | 0.08 | 0.08 | Wintergreen oil |
| Ethyl caproate | 0.01 | 0.02 | 0.02 | 0.84 | 0.70 | 1.01 | 2.23 | 1.90 | 2.12 | ND | ND | ND | 0.60 | 0.50 | 0.60 | Fruity |
| Isoamyl acetate | ND | ND | ND | 0.96 | 0.80 | 1.64 | 0.54 | 0.45 | 0.53 | 3.20 | 5.33 | 5.33 | 18.71 | 13.50 | 15.93 | Bananas |
| Benzyl acetate | ND | ND | ND | ND | ND | ND | ND | ND | ND | ND | ND | ND | 0.36 | 0.38 | 0.38 | Flowry |
| Ethyl acetate | 0.62 | 0.65 | 0.63 | 0.92 | 0.77 | 0.99 | ND | ND | ND | 514.49 | 506.07 | 508.47 | 530.49 | 529.07 | 528.47 | Fruity |
| Ethyl 3-phenylpropionate | ND | ND | ND | ND | ND | ND | 9.93 | 8.08 | 10.22 | ND | ND | ND | ND | ND | ND | Ethereal, rum, fruity, flowry |
| Isobutyl 2,2,4-trimethylpentanediol | 0.03 | 0.03 | 0.04 | 2.09 | 1.62 | 1.98 | ND | ND | ND | 0.20 | 1.52 | 0.20 | 0.10 | 0.10 | 0.10 | ND |
| Ethyl 9 - decenoate | ND | ND | ND | 27.52 | 23.16 | 25.66 | 58.20 | 33.80 | 66.54 | 0.92 | 0.76 | 1.45 | ND | ND | ND | ND |
| Propylene glycol ether acetate | ND | ND | ND | ND | ND | ND | ND | ND | ND | 0.25 | 0.20 | 0.25 | 0.11 | 0.11 | 0.11 | ND |
| Ketones |  |  |  |  |  |  |  |  |  |  |  |  |  |  |  |  |
| 2 - Undecanone | ND | ND | ND | ND | ND | ND | 0.55 | 0.44 | 0.56 | ND | ND | ND | ND | ND | ND | Citrus, oil |
| 3-Hydroxy-2-butanone | ND | ND | ND | ND | ND | ND | 0.51 | 0.41 | 0.50 | 3.75 | 3.13 | 3.75 | 24.67 | 25.94 | 25.94 | Milk |
| Phenols |  |  |  |  |  |  |  |  |  |  |  |  |  |  |  |  |
| 2,4-Ditert-butylphenol | 0.22 | 0.25 | 0.26 | 46.67 | 38.03 | 36.42 | 80.13 | 64.75 | 71.59 | 65.00 | 64.41 | 77.13 | 13.12 | 13.64 | 13.64 | ND |
| Eugenol | ND | ND | ND | ND | ND | ND | 5.15 | 3.64 | 5.89 | 1.60 | 1.27 | 1.76 | 2.53 | 2.55 | 2.55 | Clove, spicy |
| 2-Methoxy-4-vinylphenol | ND | ND | ND | ND | ND | ND | 2.74 | 2.36 | 2.86 | 1.63 | 1.36 | 1.63 | ND | ND | ND | Spicy, apple, rum, roasted peanut |
| Aldehydes |  |  |  |  |  |  |  |  |  |  |  |  |  |  |  |  |
| Acetaldehyde | ND | ND | ND | 0.45 | 0.37 | 0.45 | 4.05 | 3.46 | 4.25 | 2.91 | 2.42 | 2.91 | 2.18 | 2.20 | 2.20 | Spicy |
| Benzaldehyde | 0.02 | 0.02 | 0.02 | 1.10 | 0.87 | 1.11 | 1.95 | 1.64 | 1.85 | 12.55 | 13.42 | 14.91 | 4.78 | 5.98 | 5.98 | Almond |
| Phenyl acetaldehyde | ND | ND | ND | 1.30 | 1.08 | 1.09 | 1.40 | 1.29 | 1.78 | ND | ND | ND | ND | ND | ND | Hyacinth, cherry |
| Nonanal | 0.04 | 0.05 | 0.04 | 1.54 | 1.28 | 1.54 | 0.78 | 0.57 | 0.92 | 0.39 | 0.33 | 0.39 | 0.08 | 0.09 | 0.09 | Citrus, fatty |
| 4-Ethylbenzaldehyde | ND | ND | ND | 2.54 | 1.88 | 2.26 | ND | ND | ND | ND | ND | ND | ND | ND | ND | ND |
| Alkanes |  |  |  |  |  |  |  |  |  |  |  |  |  |  |  |  |
| Dodecylmethylc  clohexasiloxane | ND | ND | ND | 0.70 | 0.65 | 0.72 | 1.29 | 1.15 | 1.40 | ND | ND | ND | ND | ND | ND | ND |
| Cyclopentapoly dimethylsiloxane | ND | ND | ND | 3.14 | 2.56 | 3.32 | 3.03 | 2.43 | 3.04 | ND | ND | ND | ND | ND | ND | ND |
| Hexamethylcyclotrisiloxane | 0.24 | 0.25 | 0.26 | 0.71 | 0.59 | 0.71 | ND | 1.99 | 2.56 | 2.27 | 1.89 | 2.27 | 2.09 | 2.29 | 2.29 | ND |
| Hexadecane | ND | ND | ND | 1.45 | 1.21 | 1.45 | 0.76 | 0.72 | 0.78 | ND | ND | ND | ND | ND | ND | ND |
| Longifolene | ND | ND | ND | 3.57 | 2.97 | 3.57 | 4.37 | 3.52 | 4.32 | ND | ND | ND | ND | ND | ND | Woody |

^a^ ND, Not detected. Odor descriptions were cited from www.flavornet.org.


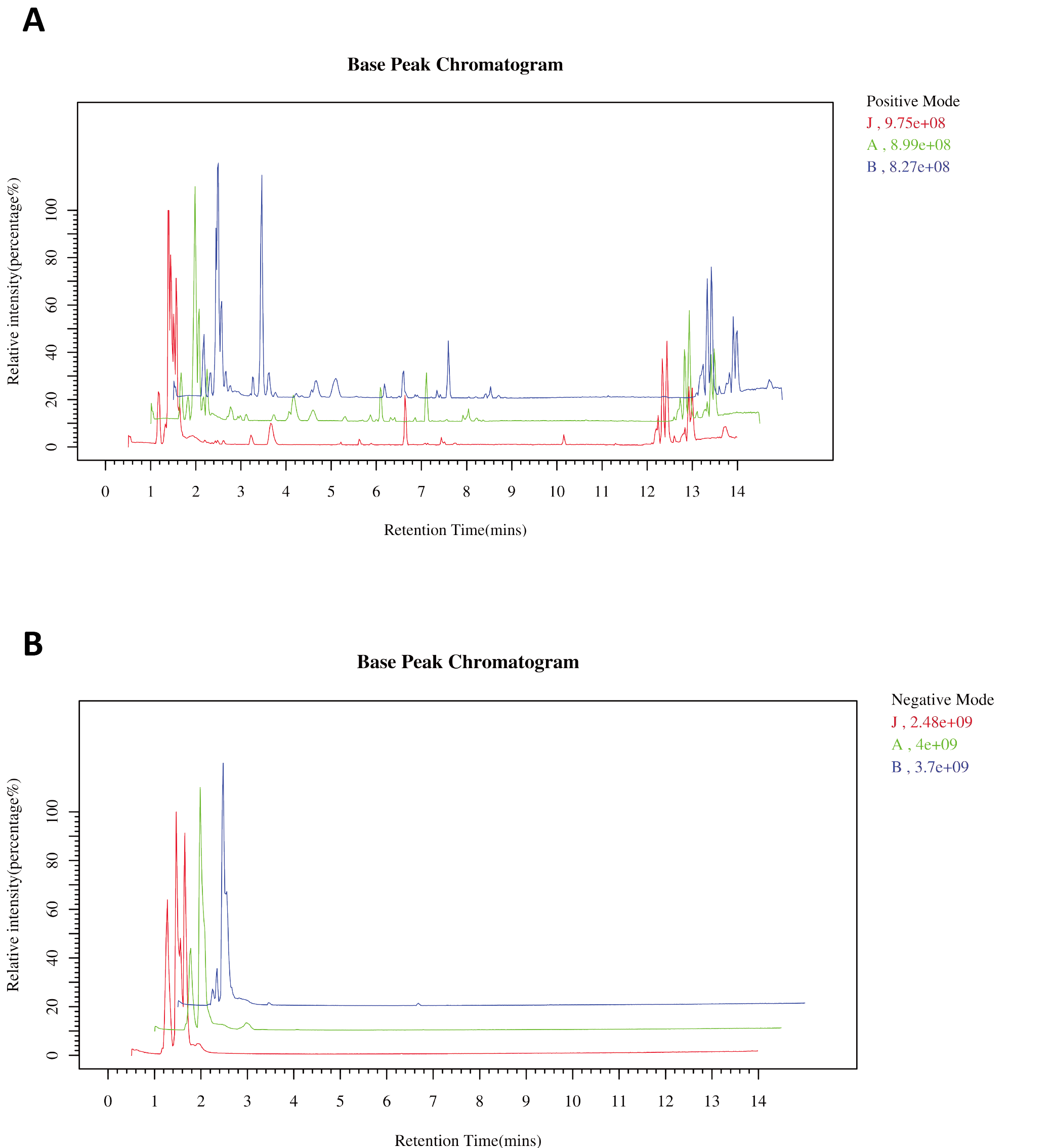


Fig. S1. Sample base peak chromatogram ( BPC ) : Positive mode (A); Negative mode (B).

**
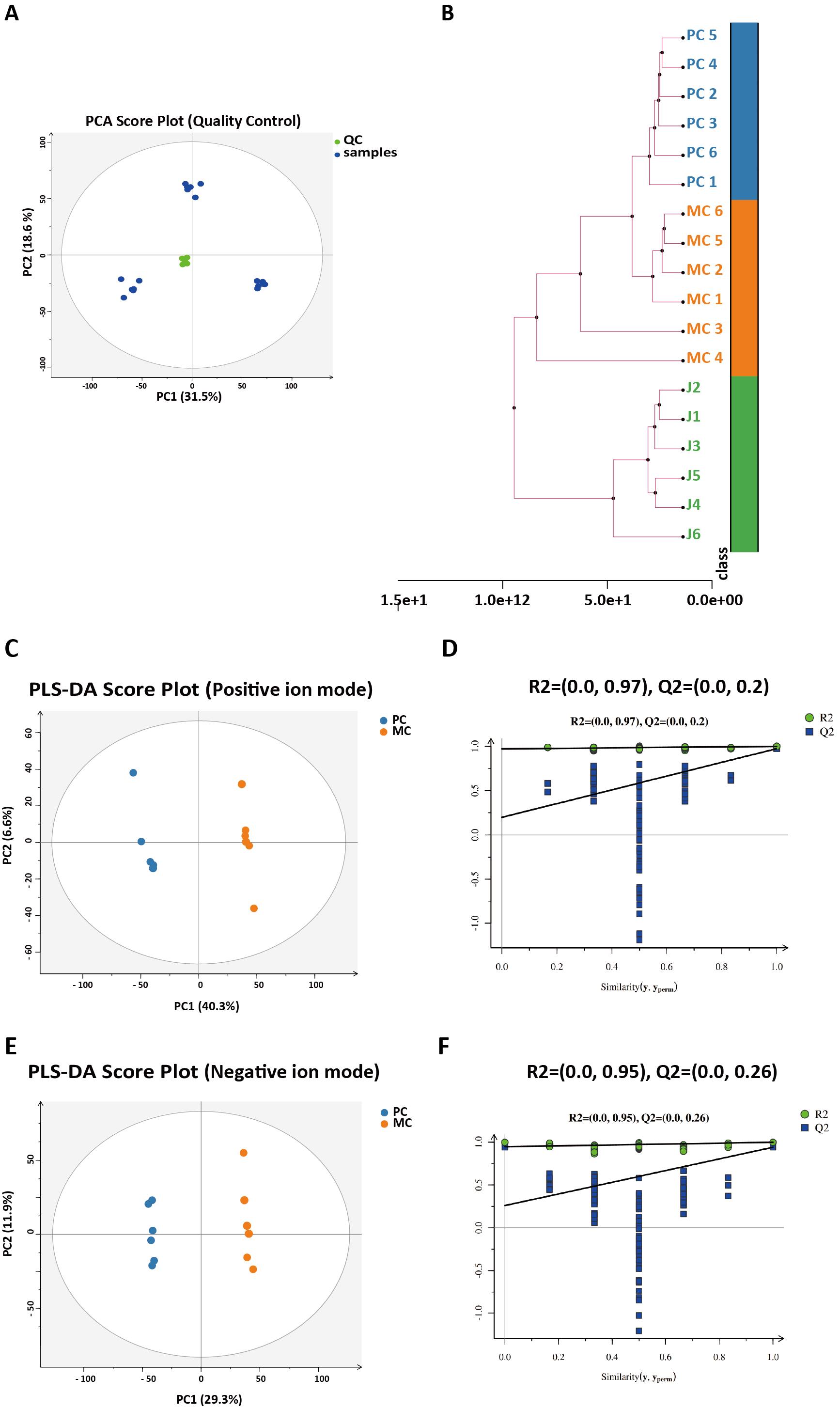
**

Fig. S2. Multivariate analysis of LC-MS based metabolomics data of apple cider vinegar with PC and MC: PCA score plots for the data of Quality Control (A) (The sample groups are color coded as follows: green = QC samples, blue = all samples); HCA clustering analysis (B); Partial least squares data analysis (PLS-DA) from positive ionization mode (C) (blue = PC, orange = MC); Permutation test result of PLS-DA from positive ionization mode (D); Partial least squares data analysis (PLS-DA) from negative ionization mode (E) (blue = PC, orange = MC); Permutation test result of PLS-DA from negative ionization mode (F).
